# Service Evaluation of the Association Between Post-Traumatic Stress Disorder and Atrial Fibrillation in Individuals Exposed to the Northern Ireland Troubles

**DOI:** 10.1192/bjo.2026.11640

**Published:** 2026-06-30

**Authors:** Finn Hughes

**Affiliations:** Queen's University Belfast, Belfast, United Kingdom

## Abstract

**Aims:**

Post-traumatic stress disorder (PTSD) is associated with autonomic nervous system and hypothalamic–pituitary–adrenal axis dysregulation, both of which are implicated in cardiovascular pathology. Individuals exposed to conflict-related trauma frequently experience long-term mental and physical health comorbidity. This service evaluation aimed to examine the prevalence and timing of atrial fibrillation (AF) among service users with PTSD who were exposed to conflict-related trauma during the Northern Ireland conflict (“The Troubles”), and to assess whether observed patterns align with established biological mechanisms linking chronic psychological stress to cardiac arrhythmia.

**Methods:**

A service evaluation was conducted using secondary analysis of fully anonymised, routinely collected health questionnaire data from service users accessing WAVE Trauma Centre, a specialist organisation supporting individuals affected by conflict-related trauma arising from the Northern Ireland conflict. Use of the data was undertaken with organisational permission from WAVE Trauma Centre. Data included demographic variables, reported PTSD diagnoses or symptoms, physical health conditions, and the temporal relationship between trauma exposure and medical diagnoses. The prevalence of atrial fibrillation among service users with PTSD was compared with published population-level prevalence estimates for Northern Ireland. Relevant neurobiological and cardiological literature was reviewed to contextualise findings.

**Results:**

Ninety-eight service users with PTSD were identified within the dataset, of whom nine had a diagnosis of atrial fibrillation (9.2%). This represents an approximately four-fold higher prevalence compared with the estimated 2.3% prevalence of atrial fibrillation in the general Northern Ireland population. In the majority of cases, atrial fibrillation was diagnosed following exposure to conflict-related trauma. A greater proportion of service users with PTSD developed atrial fibrillation before the age of 65 compared with population-based data, suggesting earlier onset. These findings are consistent with reports from large trauma-exposed cohorts demonstrating increased incidence and earlier onset of atrial fibrillation among individuals with PTSD.

**Conclusion:**

This service evaluation identifies an increased prevalence and earlier onset of atrial fibrillation among service users with PTSD following exposure to conflict-related trauma during the Northern Ireland conflict. The findings support existing evidence linking chronic stress-related neurobiological dysregulation to cardiac arrhythmogenesis. Increased awareness of cardiovascular risk within trauma-informed services may facilitate earlier detection and more integrated physical and mental healthcare for individuals with PTSD.

